# Supplementary figures and images for: Severity of infection with the SARS-CoV-2 B.1.1.7 lineage among hospitalized COVID-19 patients in Belgium
Source: PLoS One. 2022 Jun 3;17(6):e0269138. doi: 10.1371/journal.pone.0269138 (PMC9165825; doi:10.1371/journal.pone.0269138)

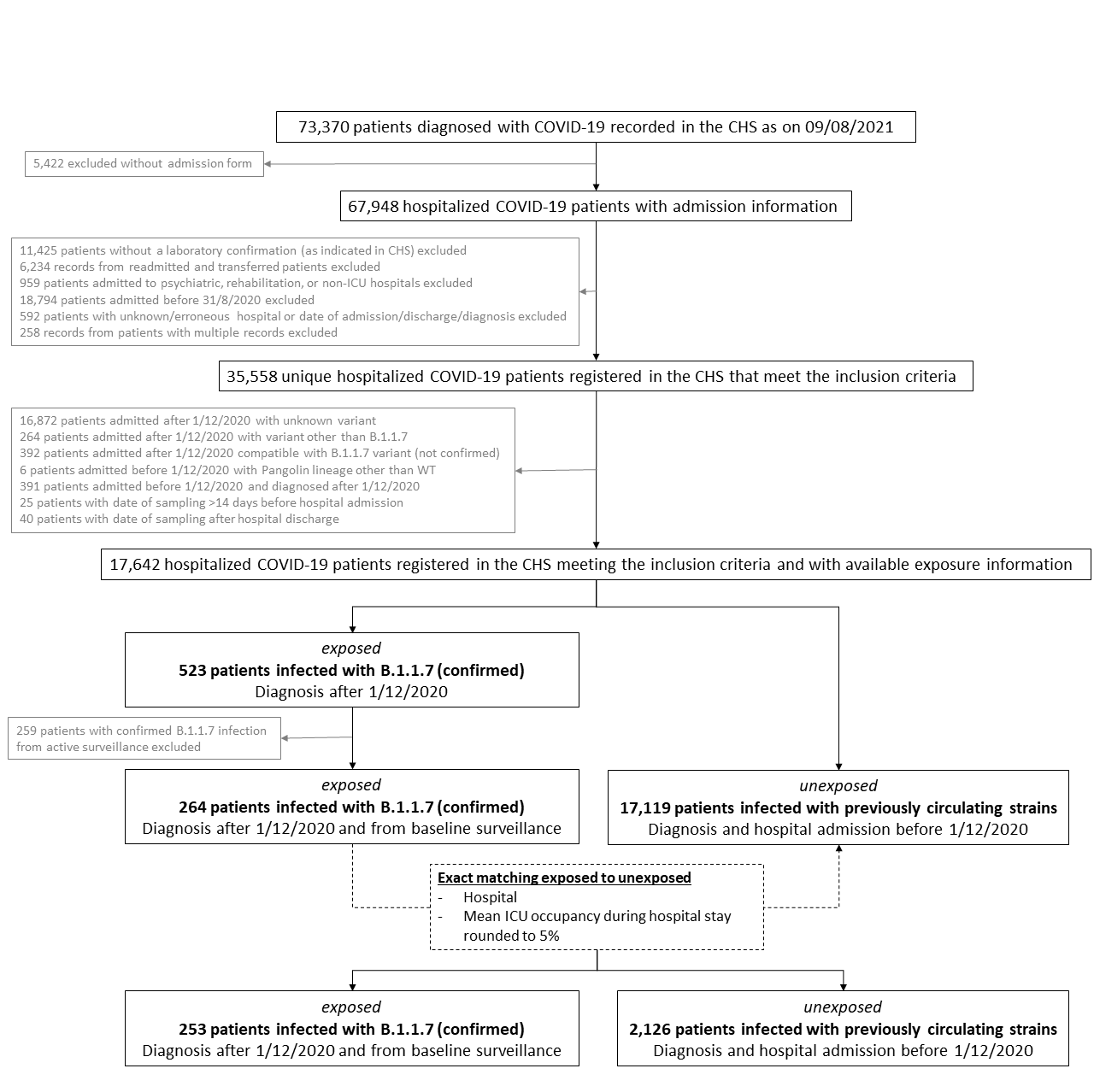

Supplement: S1 Fig — Flow chart for a sensitivity analysis within a multi-center matched cohort study to assess the impact of SARS-CoV-2 variants on COVID-19 disease severity among hospitalized patients in Belgium. (TIF) [file pone.0269138.s001.tif]

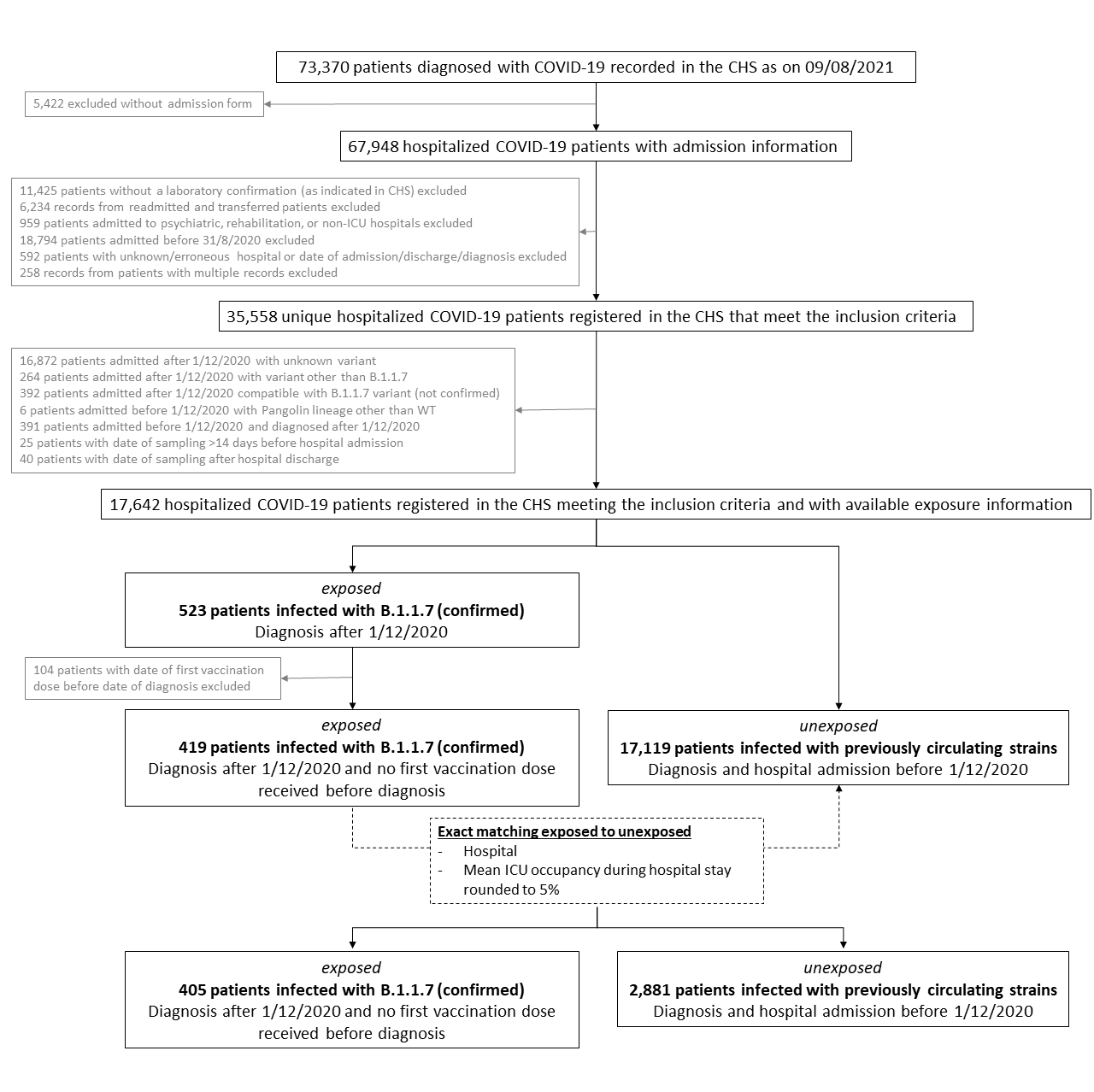

Supplement: S2 Fig — Flow chart for a sensitivity analysis within a multi-center matched cohort study to assess the impact of SARS-CoV-2 variants on COVID-19 disease severity among hospitalized patients in Belgium. (TIF) [file pone.0269138.s002.tif]
