# Supplementary material for: Severity of infection with the SARS-CoV-2 B.1.1.7 lineage among hospitalized COVID-19 patients in Belgium
Source: PLoS One. 2022 Jun 3;17(6):e0269138. doi: 10.1371/journal.pone.0269138 (PMC9165825; doi:10.1371/journal.pone.0269138)
Supplement: S1 Table — Results (overall and stratified per age group) for a sensitivity analysis within a multi-center matched cohort study to assess the impact of SARS-CoV-2 variants on COVID-19 disease severity among hospitalized patients in Belgium. (DOCX) [file pone.0269138.s003.docx]

**Supplementary Table 1. Risk per exposure group (in %), Relative Risk (RR) and Risk Difference (RD, in %) estimates and 95% Confidence Interval (CI) for main and secondary outcomes, overall and stratified per age group, for the sensitivity analysis when only considering Whole-Genome Sequencing (WGS) results obtained through baseline surveillance within a multi-center matched cohort study to assess the impact of SARS-CoV-2 variants on COVID-19 disease severity among hospitalized patients in Belgium.**

| Outcome | Risk (in %) ^a^ [95% CI] | | RR [95% CI] | RD (in %) [95% CI] |
| --- | --- | --- | --- | --- |
|  | **PCV** | **B.1.1.7 ^b^** |  |  |
| **Overall** | | | | |
| Severe COVID-19 ^c^ | 25.6 [22.7 – 28.6] | 31.3 [23.1 – 39.5] | 1.22 [0.86 – 1.58] | 5.7 [-3.2 – 14.5] |
| ICU-admission | 14.8 [12.5 – 17.0] | 25.6 [18.3 – 32.8] | 1.73 [1.17 – 2.29] | 10.8 [3.2 – 18.4] |
| In-hospital mortality | 16.5 [13.9 – 19.0] | 13.4 [5.1 – 21.7] | 0.82 [0.29 – 1.34] | -3.0 [-11.6 – 5.6] |
| **Age ≤ 65 years** | | | | |
| Severe COVID-19 ^c^ | 14.6 [11.6 – 17.5] | 25.7 [17.7 – 33.7] | 1.76 [1.12 – 2.40] | 11.1 [2.7 – 19.5] |
| ICU-admission | 13.2 [10.4 – 16.0] | 24.5 [16.5 – 32.5] | 1.86 [1.17 – 2.55] | 11.3 [3.0 – 19.6] |
| In-hospital mortality | 4.1 [2.6 – 5.7] | 6.4 [0.0 – 12.8] | 1.54 [0.00 – 3.42] | 2.2 [-4.5 – 8.9] |
| **Age > 65 years** | | | | |
| Severe COVID-19 ^c^ | 34.5 [27.3 – 41.6] | 39.6 [24.6 – 54.5] | 1.15 [0.45 – 1.85] | 5.1 [-11.9 – 22.0] |
| ICU-admission | 16.2 [9.4 – 23.0] | 30.1 [13.6 – 46.6] | 1.86 [0.00 – 7.74] | 13.9 [-4.2 – 32.0] |
| In-hospital mortality | 26.1 [21.0 – 31.1] | 22.1 [7.5 – 36.8] | 0.85 [0.11 – 1.59] | -3.9 [-19.6 – 11.8] |

**Notes:**

^a^ Standardized risk with respect to the model and covariate distribution.

^b^ Confirmed via Whole-Genome Sequencing (WGS) and obtained through baseline surveillance

^c^ Presence of acute respiratory distress syndrome (ARDS), ICU admission and/or in-hospital death.

**Abbreviations:** CI, confidence interval; ICU, intensive care unit; PCV, previously circulating variants; RD, risk difference; RR, risk ratio.
