# Supplementary material for: Severity of infection with the SARS-CoV-2 B.1.1.7 lineage among hospitalized COVID-19 patients in Belgium
Source: PLoS One. 2022 Jun 3;17(6):e0269138. doi: 10.1371/journal.pone.0269138 (PMC9165825; doi:10.1371/journal.pone.0269138)
Supplement: S2 Table — Results (overall and stratified per age group) for a sensitivity analysis within a multi-center matched cohort study to assess the impact of SARS-CoV-2 variants on COVID-19 disease severity among hospitalized patients in Belgium. (DOCX) [file pone.0269138.s004.docx]

**Supplementary Table 2. Risk per exposure group (in %), Relative Risk (RR) and Risk Difference (RD, in %) estimates and 95% Confidence Interval (CI) for main and secondary outcomes, overall and stratified per age group, for the sensitivity analysis excluding patients that had received a first vaccination dose before their COVID-19 diagnosis, within a multi-center matched cohort study to assess the impact of SARS-CoV-2 variants on COVID-19 disease severity among hospitalized patients in Belgium.**

| Outcome | Risk (in %) ^a^ [95% CI] | | RR [95% CI] | RD (in %) [95% CI] |
| --- | --- | --- | --- | --- |
|  | **PCV** | **B.1.1.7 ^b^** |  |  |
| **Overall** | | | | |
| Severe COVID-19 ^c^ | 26.9 [24.3 – 29.6] | 30.6 [24.6 – 36.0] | 1.14 [0.89 – 1.38] | 3.7 [-2.8 – 10.1] |
| ICU admission | 15.6 [13.3 – 17.9] | 21.6 [16.6 – 26.8] | 1.39 [0.99 – 1.79] | 6.1 [0.4 – 11.7] |
| In-hospital mortality | 15.9 [13.6 – 18.2] | 15.1 [8.6 – 21.7] | 0.95 [0.53 – 1.38] | - 0.7 [-7.4 – 5.9] |
| **Age ≤ 65 years** | | | | |
| Severe COVID-19 ^c^ | 16.4 [13.9 – 18.9] | 28.0 [21.7 – 34.3] | 1.70 [1.26 – 2.15] | 11.6 [5.0 – 18.1] |
| ICU admission | 14.1 [11.6 – 16.6] | 26.4 [20.1 – 32.8] | 1.88 [1.33 – 2.43] | 12.3 [5.7 – 19.0] |
| In-hospital mortality | 3.8 [2.5 – 5.2] | 8.5 [3.1 – 14.0] | 2.22 [0.65 – 3.79] | 4.7 [-0.8 – 10.2] |
| **Age > 65 years** | | | | |
| Severe COVID-19 ^c^ | 34.8 [30.8 – 38.7] | 34.8 [23.7 – 45.8] | 1.00 [0.65 – 1.35] | 0.0 [-11.8 – 11.8] |
| ICU admission | 16.8 [12.6 – 20.9] | 23.4 [14.4 – 32.5] | 1.40 [0.48 – 2.31] | 6.7 [-3.4 – 16.7] |
| In-hospital mortality | 24.5 [20.4 – 28.5] | 25.3 [13.0 – 37.5] | 1.03 [0.39 – 1.67] | 0.8 [-12.0 – 13.6] |

**Notes:**

^a^ Standardized risk with respect to the model and covariate distribution.

^b^ Confirmed via Whole-Genome Sequencing (WGS) and obtained through baseline surveillance

^c^ Presence of acute respiratory distress syndrome (ARDS), ICU admission and/or in-hospital death.

**Abbreviations:** CI, confidence interval; ICU, intensive care unit; PCV, previously circulating variants; RD, risk difference; RR, risk ratio.
