# Supplementary material for: Severity of infection with the SARS-CoV-2 B.1.1.7 lineage among hospitalized COVID-19 patients in Belgium
Source: PLoS One. 2022 Jun 3;17(6):e0269138. doi: 10.1371/journal.pone.0269138 (PMC9165825; doi:10.1371/journal.pone.0269138)
Supplement: S3 Table — Sensitivity analysis within a multi-center matched cohort study to assess the impact of SARS-CoV-2 variants on COVID-19 disease severity among hospitalized patients in Belgium. (DOCX) [file pone.0269138.s005.docx]

**Supplementary Table 3. Sensitivity analysis using the E-value within a multi-center matched cohort study to assess the impact of SARS-CoV-2 variants on COVID-19 disease severity among hospitalized patients in Belgium.**

| **Outcome** | **Observed RR and 95% CI** | **E-value** | **E-value lower limit CI** | **E-value upper limit CI** | **Multi-bias E-value^a^** |
| --- | --- | --- | --- | --- | --- |
| Severe COVID-19^b^ | 1.15 [0.93 – 1.38] | 1.57 | 1 | - | 1.35 |
| ICU admission | 1.36 [1.03 – 1.68] | 2.06 | 1.21 | - | 1.60 |
| In-hospital mortality | 0.92 [0.62 – 1.23] | 1.39 | - | 1 | 1.25 |

**Notes:**

^a^ Multi-bias E-value for unmeasured confounding and selection bias.

^b^ Presence of acute respiratory distress syndrome (ARDS), ICU admission and/or in-hospital death.

**Abbreviations:** CI, confidence interval; ICU, intensive care unit; RR, risk ratio.
