# Supplementary material for: Severity of infection with the SARS-CoV-2 B.1.1.7 lineage among hospitalized COVID-19 patients in Belgium
Source: PLoS One. 2022 Jun 3;17(6):e0269138. doi: 10.1371/journal.pone.0269138 (PMC9165825; doi:10.1371/journal.pone.0269138)
Supplement: S4 Table — Baseline characteristics between hospitalized patients in Belgium admitted after March 1st 2021 with available variant information (confirmed) obtained through baseline surveillance and without available variant information. (DOCX) [file pone.0269138.s006.docx]

**Supplementary Table 4. Baseline characteristics between hospitalized patients in Belgium admitted after March 1^st^ 2021 with available variant information (confirmed) obtained through baseline surveillance and without available variant information.**

|  | Hospitalized patients admitted after 1/3/2021 and with available variant information (confirmed) from baseline surveillance (n = 357) | | | Hospitalized patients admitted after 1/3/2021 and without available variant information (n = 8,927) | | |
| --- | --- | --- | --- | --- | --- | --- |
|  |  |  |  |  |  |  |
|  |  | **%** | **n** |  | **%** | **n** |
| Demographics | | | | | | |
| Age (years), median (IQR) | 60 (47-73) |  | 357 | 62 (49-76) |  | 8923 |
| Male gender, n (%) | 214 | 59.9 | 357 | 4724 | 53.0 | 8921 |
| Nursing home resident, n (%) | 18 | 5.1 | 354 | 207 | 2.3 | 8846 |
| Ethnicity, n (%) | | | | | | |
| European | 272 | 84.0 | 324 | 6942 | 84.9 | 8173 |
| North-African | 26 | 8.0 | 324 | 683 | 8.4 | 8173 |
| Sub-Saharan African | 13 | 4.0 | 324 | 195 | 2.4 | 8173 |
| Asian | 10 | 3.1 | 324 | 237 | 2.9 | 8173 |
| Hispanic | 3 | 0.9 | 324 | 86 | 1.1 | 8173 |
| Comorbidities | | | | | | |
| Cardiovascular Disease, n (%) | 92 | 25.8 | 356 | 2177 | 24.5 | 8900 |
| History of Arterial Hypertension, n (%) | 125 | 35.1 | 356 | 3046 | 34.2 | 8900 |
| Diabetes mellitus, n (%) | 84 | 23.6 | 356 | 1842 | 20.7 | 8900 |
| Obesity, n (%) | 60 | 16.9 | 356 | 1572 | 17.7 | 8900 |
| Chronic Pulmonary Disease, n (%) | 52 | 14.6 | 356 | 1139 | 12.8 | 8900 |
| Chronic Neurological Disease, n (%) | 17 | 4.8 | 356 | 536 | 6.0 | 8900 |
| Chronic Cognitive Deficit, n (%) | 16 | 4.5 | 356 | 461 | 5.2 | 8900 |
| Chronic Renal Disease, n (%) | 43 | 12.1 | 356 | 907 | 10.2 | 8900 |
| Chronic Liver Disease, n (%) | 10 | 2.8 | 356 | 230 | 2.6 | 8900 |
| Solid Cancer, n (%) | 30 | 8.4 | 356 | 734 | 8.2 | 8900 |
| Haematological Cancer, n (%) | 10 | 2.8 | 356 | 141 | 1.6 | 8900 |
| Chronic Immunosuppression, n (%) | 13 | 3.7 | 356 | 171 | 1.9 | 8900 |
| Socio-economic status | | | | | | |
| Education level^a^, n (%) |  |  |  |  |  |  |
| Lower | 43 | 20.0 | 215 | 1328 | 23.9 | 5566 |
| Lower secondary | 58 | 27.0 | 215 | 1650 | 29.6 | 5566 |
| Higher secondary | 66 | 30.7 | 215 | 1585 | 28.5 | 5566 |
| Post-secondary higher education | 48 | 22.3 | 215 | 1003 | 18.0 | 5566 |
|  |  |  |  |  |  |  |
| Population density^b^, median (IQR) | 780 (330-2100) |  | 354 | 830 (380-2000) |  | 8104 |
| Median taxable income per capita^c^, median (IQR) | 27000 (24000-29000) |  | 354 | 27000 (24000-28000) |  | 8104 |
| Exposure | | | | | | |
| Place of infection, n (%) | | | | | | |
| Community-acquired | 317 | 89.5 | 354 | 8278 | 94.1 | 8797 |
| Hospital-acquired^d^ | 24 | 6.8 | 354 | 337 | 3.8 | 8797 |
| Nursing home-acquired | 13 | 3.7 | 354 | 182 | 2.1 | 8797 |
| Vaccination status | | | | | | |
| Vaccination category^e^, n (%) | | | | | | |
| Pre-vaccination | 298 | 83.5 | 357 | 7784 | 90.2 | 8628 |
| Partial vaccination | 38 | 10.6 | 357 | 646 | 7.5 | 8628 |
| Post-vaccination | 21 | 5.9 | 357 | 197 | 2.3 | 8628 |
| Disease characteristics | | | | | | |
| Fever at admission, n (%) | 180 | 50.7 | 355 | 4240 | 48.0 | 8836 |
| Viral syndrome at admission, n (%) | 156 | 43.9 | 355 | 4062 | 46.0 | 8836 |
| Lower respiratory symptoms at admission, n (%) | 268 | 75.5 | 355 | 6195 | 70.1 | 8836 |
| Upper respiratory symptoms at admission, n (%) | 34 | 9.6 | 355 | 819 | 9.3 | 8836 |
| Gastrointestinal symptoms at admission, n (%) | 95 | 26.8 | 355 | 2535 | 28.7 | 8836 |
| Anosmia at admission, n (%) | 31 | 8.7 | 355 | 689 | 7.8 | 8836 |
| CRP (mg/l) on admission, median (IQR) | 68 (29-130) |  | 335 | 62 (25-120) |  | 8282 |
| Lymphocytes (/mm^3^) on admission, median (IQR) | 770 (400-1200) |  | 319 | 890 (530-1400) |  | 8061 |
| LDH (U/l) on admission, median (IQR) | 340 (250-470) |  | 295 | 340 (260-460) |  | 7517 |
| PaO_2_ (mmHg) on admission, median (IQR) | 66 (58-75) |  | 239 | 65 (58-74) |  | 5967 |
| Hospital characteristics | | | | | | |
| Type of hospital, n (%) | | | | | | |
| General hospital | 230 | 64.4 | 357 | 6427 | 72.0 | 8927 |
| General hospital with university character | 59 | 16.5 | 357 | 1645 | 18.4 | 8927 |
| University hospital | 68 | 19.0 | 357 | 855 | 9.6 | 8927 |
| Mean ICU occupancy during hospital stay^f^, median (IQR) | 33 (19-46) |  | 357 | 41 (30-51) |  | 8927 |
| Outcomes | | | | | | |
| Severe^g^ COVID-19, n (%) | 101 | 28.7 | 352 | 2222 | 25.2 | 8822 |
| ICU transfer, n (%) | 78 | 22.0 | 355 | 1395 | 15.6 | 8918 |
| General hospital | 48 | 20.9 | 230 | 878 | 13.7 | 6424 |
| General hospital with university character | 11 | 19.0 | 58 | 301 | 18.3 | 1644 |
| University hospital | 19 | 28.4 | 67 | 216 | 25.4 | 850 |
| In-hospital mortality, n (%) | 45 | 12.7 | 354 | 1139 | 12.9 | 8814 |
| Invasive ventilation, n (%) | 26 | 7.3 | 357 | 490 | 5.5 | 8920 |
| ECLS, n (%) | 0 | 0.0 | 357 | 35 | 0.4 | 8924 |
| Hospital length of stay (days), median (IQR) | 7 (4-15) |  | 357 | 7 (4-13) |  | 8927 |

**Notes:**

^a^ Highest degree obtained. ED1: lower; ED2: lower secondary; ED3: higher secondary; ED5: higher.

^b^ Population density at the postal code level of the residence of the patient.

c Median net taxable income per capita at the postal code level of the residence of the patient.

d Symptom onset or diagnosis more than 8 days after hospital admission.

^e^ Pre-vaccination: diagnosed when no dose received or before 14 days after the first dose (for Pfizer/BioNTech, AstraZeneca and Moderna vaccine); Partial vaccination: diagnosed 14 days after the first dose (for Pfizer/BioNTech, AstraZeneca and Moderna vaccine) but before 14 days after the full dose (2 doses for Pfizer/BioNTech, AstraZeneca and Moderna vaccine and 1 dose for Johnson & Johnson vaccine); Post-vaccination: diagnosed ≥14 days after the full dose (2 doses for Pfizer/BioNTech, AstraZeneca and Moderna vaccine and 1 dose for Johnson & Johnson vaccine).

^f^ The mean of the number of ICU COVID-19 patients in the hospital divided by the number of recognized ICU beds reserved for COVID-19 patients calculated over the hospital length of stay of the patient

^g^ Defined as a combination of three binary severity indicators: having been admitted to ICU or developed acute respiratory distress syndrome (ARDS) and/or died in the hospital.

**Abbreviations:** CRP, C-reactive protein; ECLS, Extracorporeal life support; ICU, intensive care unit; IQR, inter-quartile range; LDH, lactate dehydrogenase; PaO_2_, partial blood oxygen pressure
